# Size Matters: Individual Variation in Ectotherm Growth and Asymptotic Size

**DOI:** 10.1371/journal.pone.0146299

**Published:** 2016-01-05

**Authors:** Richard B. King, Kristin M. Stanford, Peter C. Jones, Kent Bekker

**Affiliations:** 1 Department of Biological Sciences, Northern Illinois University, DeKalb, Illinois, United States of America; 2 F. T. Stone Laboratory, The Ohio State University, Put-in-Bay, Ohio, United States of America; 3 Department of Herpetology, The Toledo Zoo, Toledo, Ohio, United States of America; CSIC-EEZA, SPAIN

## Abstract

Body size, and, by extension, growth has impacts on physiology, survival, attainment of sexual maturity, fecundity, generation time, and population dynamics, especially in ectotherm animals that often exhibit extensive growth following attainment of sexual maturity. Frequently, growth is analyzed at the population level, providing useful population mean growth parameters but ignoring individual variation that is also of ecological and evolutionary significance. Our long-term study of Lake Erie Watersnakes, *Nerodia sipedon insularum*, provides data sufficient for a detailed analysis of population and individual growth. We describe population mean growth separately for males and females based on size of known age individuals (847 captures of 769 males, 748 captures of 684 females) and annual growth increments of individuals of unknown age (1,152 males, 730 females). We characterize individual variation in asymptotic size based on repeated measurements of 69 males and 71 females that were each captured in five to nine different years. The most striking result of our analyses is that asymptotic size varies dramatically among individuals, ranging from 631–820 mm snout-vent length in males and from 835–1125 mm in females. Because female fecundity increases with increasing body size, we explore the impact of individual variation in asymptotic size on lifetime reproductive success using a range of realistic estimates of annual survival. When all females commence reproduction at the same age, lifetime reproductive success is greatest for females with greater asymptotic size regardless of annual survival. But when reproduction is delayed in females with greater asymptotic size, lifetime reproductive success is greatest for females with lower asymptotic size when annual survival is low. Possible causes of individual variation in asymptotic size, including individual- and cohort-specific variation in size at birth and early growth, warrant further investigation.

## Introduction

Scientific inquiry often begins with an incidental observation. The impetus for the research reported here was an impression from a long-term capture-mark-recapture study of Lake Erie Watersnakes that (1) individuals caught repeatedly over many years appeared not to have grown and (2) the size at which growth ceased varied dramatically from individual to individual. These observations seemed at odds with depictions of reptilian growth, including our own [1–3], which imply that individuals grow continuously, albeit at reduced rates, toward a common asymptote. Such depictions, often illustrated by a single curvilinear relationship between age and size, seemed not to account for the cessation of growth and, especially, the variation in adult size that we were observing. Thus, we sought to quantify long-term growth patterns of individual Lake Erie Watersnakes and test whether asymptotic size varied among individuals.

There is no dearth of analyses of reptilian growth. As of Dec 2015, Andrews’ review [4] had been cited 503 times (Google Scholar) and a Web of Science search for “reptile growth” returned more than 1,000 citations. This is for good reason; body size, and, by extension, growth has impacts on physiology, survival, attainment of sexual maturity, fecundity, generation time, and population dynamics, especially in ectotherm animals that often exhibit extensive growth following attainment of sexual maturity [5–9]. Frequently, growth is analyzed at the population level and results in population mean growth parameters. Such parameters are useful for estimating age at reproductive maturity [2, 10], testing hypotheses related to sexual size dimorphism [11], and making comparisons between sexes or among years, populations, and species (e.g., [12–14]). However, they ignore individual variation in growth trajectories that is also likely to have ecological and evolutionary significance. For example, studies of water pythons [15] and meadow vipers [16] demonstrate cohort-specific patterns of neonatal growth due to year-to-year variation in prey abundance or offspring condition, with consequences for growth and survival later in life.

Recognition of individual variation in growth is seen in analyses of Australian freshwater crocodiles in which slow, average, and rapidly growing individuals were analyzed separately [17] and by the superimposition of individual growth on population mean growth trajectories (e.g., Fig 1 in [18]; Fig 1 in [16]; Fig 6 in [19]). However, formal analyses of individual growth in reptiles are rare, perhaps in part because the necessary sample size and study duration is difficult to achieve in rare and long-lived species. Our study of the Lake Erie Watersnake, *Nerodia sipedon insularum*, provides an exception to this pattern. Following listing of the Lake Erie Watersnake as threatened under the Federal Endangered Species Act due to declining population size and geographic distribution restricted to islands in western Lake Erie [20], we began annual population censuses in which large numbers of animals were individually marked and subsequently recaptured. Here, we use these data to document individual variation in asymptotic size based on growth trajectories of animals captured in five or more years. Because these animals were adults of unknown age when first captured, fitting unconstrained growth models in which all growth parameters are free to vary can produce unrealistic parameter estimates. In particular, the lack of data on younger, more rapidly growing age classes results in spurious estimates of the growth rate constant *k*. Consequently, we first characterized population mean growth in two ways; (1) by analysis of sizes of animals of known age and (2) by analysis of growth increments from repeated captures of animals of unknown age. We then used those results to fix the value of *k* in our analysis of individual growth and asymptotic size. As a result, we provide a comprehensive analysis of growth in Lake Erie Watersnakes of all ages based on more than 18,000 captures spanning 13 years. Further, we characterize individual variation in asymptotic size based on 63 males and 34 females that were captured and measured in each of five or more years and discuss potential causes and consequences of this variation.

## Methods

The Lake Erie Watersnake is a medium sized New World natricine snake allied with other watersnakes, gartersnakes, and related taxa [21]. Its distribution is restricted to the island region of Western Lake Erie where it inhabits rocky shorelines and forages mostly for benthic fishes in nearshore waters [1]. It is active from April to October, courtship and mating occur in spring, and live young are born in late summer [1]. Lake Erie Watersnakes were hand captured and individually marked at multiple study sites on islands in western Lake Erie from 1980 onward [1, 22]. Data analyzed here were collected from 2000–2012 during annual population censuses encompassing 14 study sites, including both public and private lands, on five islands as mandated in the Lake Erie Watersnake recovery plan [23] and post-delisting monitoring plan [24]. Watersnakes were captured during area-constrained searches, measured to obtain snout-vent length (SVL), weighed, and classified by sex. Snakes greater than ca. 300 mm SVL were individually marked via subcutaneous injection of Passive Integrated Transponders (PIT tags). All snakes were released at their site of capture shortly after processing.

In some years, near-term gravid females were held in captivity until parturition (ca. 1–3 weeks) to provide data on reproductive characteristics and allow marking of neonates [25]. Females were housed in 40 liter aquaria containing a shelter and water dish. A thermal gradient was provided using heat tape under one end of aquaria. Females that were in captivity for more than two weeks were offered fish, but most refused food. Following birth, neonates were group housed by litter with access to water and shelter for up to two weeks until data collection and marking was completed. Neonates were batch marked via subcutaneous injection of magnetic wire tags (Northwest Marine Technologies) and released at their mothers’ site of capture (n = 1276 neonates from 49 litters in 2003; 399 neonates from 19 litters in 2004; 639 neonates from 23 litters in 2007, 1330 neonates from 60 litters in 2009). Upon recapture, wire-tagged animals were individually marked by scale clipping [1] or with PIT tags.

Protocols regarding the use of live vertebrate animals were approved by the Northern Illinois University Institutional Animal Care and Use Committee (ORC 238, ORC 282, ORC 326, LA12-003) and The Ohio State University Institutional Animal Care and Use Committee (2004A0048, 2007A0046, 2010A00000148). During the duration of this study, the Lake Erie Watersnake was protected as threatened under the U.S. Federal Endangered Species Act and as endangered by the Ohio Department of Natural Resources [20]. Research described here was carried out under permits from the U. S. Fish and Wildlife Service and the Ohio Division of Wildlife. Permission to work on public lands was granted by the Ohio Department of Natural Resources; permission to work on private lands was granted by individual land owners.

### Age-class and Cohort Assignment

**Fig 1:**
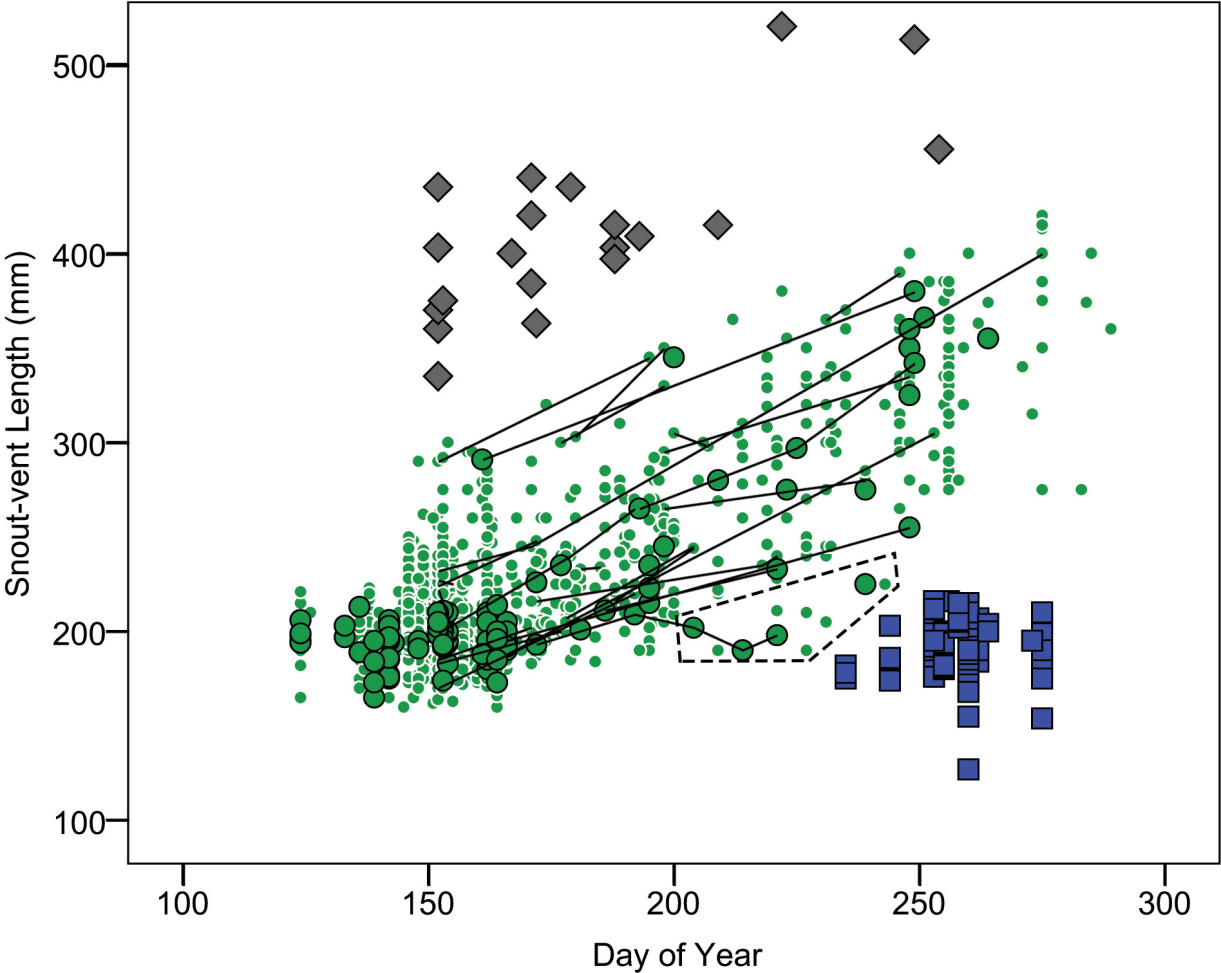
Relationship between capture day of year (DOY) and SVL for Lake Erie Watersnakes. Symbols distinguish members of age-class 0 (squares), wire-tagged (circles with black outline) and free-ranging (circles without outline) members of age class 1, and wire-tagged members of age-class 2 (diamonds). Lines show within-year growth trajectories for age-class 1 individuals that were captured multiple times within a year. Dashed polygon highlights members of age-class 1 that showed evidence of failure to thrive.

Age-class (year of life) and cohort (year of birth) membership could be assigned with confidence to two sets of animals, (1) neonates born to wild-caught females and marked with wire tags and (2) free-ranging animals captured in their first (age class 0) or second (age class 1) active season. Free-ranging animals captured in their first or second active season were identified by plotting snake SVL versus capture day of year (DOY) separately for each year of the study (See S1 Fig). The earliest date of parturition among wild-caught females was 24 August (DOY = 236) and the largest offspring produced measured 224 mm SVL. Using these criteria as a guide, animals captured on or after 15 August and measuring 225 mm SVL or less were classified as members of age-class 0. Members of age-class 1 could be identified as a largely discrete group that in spring were similar in size to members of age-class 0 and that followed a trajectory of increasing size thereafter (Fig 1). Classification of these animals as age-class 1 was corroborated with data on wire tagged snakes known to belong to age-class 1 and growth trajectories of animals captured multiple times within a year (Fig 1). Subsequent recaptures of animals of known age and cohort provided size-at-age data through age-class 9.

### Population Mean Growth Trajectories

**Fig 2:**
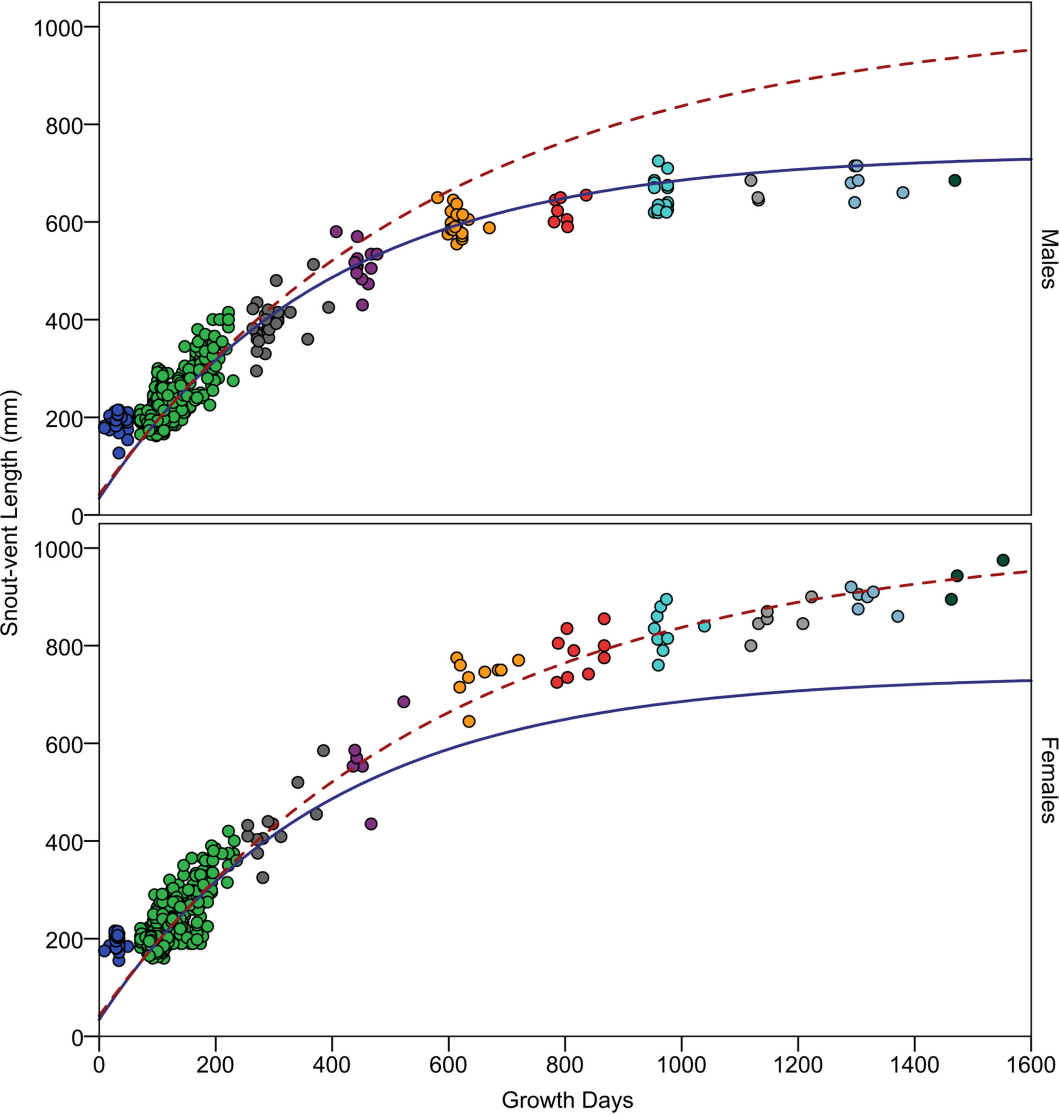
Growth trajectories of male and female Lake Erie Watersnakes. Known-age males (upper) and females (lower) are indicated by points; colors distinguish age-classes 0 (left) through 9 (right). Composite Von Bertalanffy growth functions, based on parameter estimates from analyses of animals of both known and unknown age, are indicated by solid (males) and dashed (females) lines (males: *SVL*_*t*_ = 740 − ((740 − 44)*e*^−0.0026*t*^); females: *SVL*_*t*_ = 1018 − ((1018 − 51)*e*^−0.0017*t*^)). Because temperate zone snakes do not grow during hibernation, age is measured in growth days, an estimate of age in days excluding hibernation (see text).

We characterized population mean growth trajectories in two ways; (1) by analysis of sizes of animals of known age and (2) by analysis of growth increments from repeated captures of animals of unknown age. Analysis of each data set is likely to be biased in different ways [26]. Our data on known age animals consist predominantly of members of age-class 1 with data on decreasing numbers of individuals belonging to age-class 2 through 9 (Fig 2). Furthermore, recaptures of the same individuals in successive years means that a few individuals are disproportionately represented in the oldest age classes. Consequently, these data are likely to provide relatively unbiased estimates of growth early in life, as characterized by the growth rate constant *k*, but biased estimates of growth later in life, as characterized by asymptotic size *SVL*_*A*_ [26]). In contrast, our data on growth increments of animals of unknown age consists predominantly of larger (hence older) individuals (S2 Fig). Consequently, these data are likely to provide biased estimates of the growth early in life, but unbiased estimates of growth later in life [26]). To address this problem, we analyzed growth of animals of known age with the goal of estimating the growth constant, *k*. We then used the resulting value of *k* in our analyses of growth of animals of unknown age, with the goal of estimating population mean asymptotic size, *SVL*_*A*_.

For animals of known age, we fit the three-parameter von Bertalanffy growth equation

$$SVL_{t}=SVL_{A}-((SVL_{A}-SVL_{0})e^{-kt})$$ (1)

where *SVL*_*t*_ is length at age *t*, *SVL*_*A*_ is the population mean asymptotic length, *SVL*_*0*_ is length at time 0, *k* is a growth rate constant and *t* is age. We generated estimates of *SVL*_*A*_, *SVL*_*0*_, and *k* separately for males and females using an iterative non-linear regression procedure in IBM SPSS Statistics 21. We followed [27] in estimating *SVL*_*0*_ directly rather than substituting size at birth in order to minimize bias in estimating the growth rate constant, *k*.

Because temperate-zone snakes do not grow during hibernation [28], we measured age in “growth days,” an estimate of the number of days of growth during the active season that a given snake had experienced. Based on dates of hibernation ingress and egress observed using radio-telemetry, we assumed that growth was restricted to the 185 day period from 27 April– 17 October [29]. We further assumed that birth occurred on 15 August (the earliest date of parturition among wild-caught females was 24 August). Variation in dates of birth and hibernation ingress and egress among individuals introduces error into our estimates of age, but this error should be relatively small, especially for age-classes ≥ 1 upon which our analyses are focused.

For animals of unknown age, we characterized growth using measures of SVL for animals captured in two successive years; within-season growth increments and growth increments spanning multiple years were excluded. We then fit the von Bertalanffy growth model using Fabens’ method [30]

$$SVL_{2}=SVL_{A}-((SVL_{A}-SVL_{1})e^{-kt})$$ (2)

where *SVL*_*1*_ and *SVL*_*2*_ are observed lengths in successive years, *SVL*_*A*_ is the population mean asymptotic length, *k* is a growth rate constant and *t* is age in growth days. As above, we assumed that growth was restricted to 27 April– 17 October. Furthermore, we fixed *k* to the sex-specific values obtained from our analysis of animals of known age. We generated estimates of *SVL*_*A*_ separately for males and females using an iterative non-linear regression procedure in IBM SPSS Statistics 21. Although *SVL*_*0*_ does not appear in Eq (2), its value can be obtained analytically by substituting *SVL*_*A*_ from Eq (2) and SVL at a known age, *t*, into Eq (1). We used SVL at birth (189.65 mm, the mean of 3,776 live-born offspring from 142 litters) and *t* = 92 (the age in growth days on which SVL = 189.65 from our analysis of known-age individuals).

From 2000 to 2012, we accumulated 18,147 capture records of 12,491 individual watersnakes of known sex and SVL at 14 intensive study sites on five islands (9,928 captures of 7,013 males, 7,471 captures of 5,478 females). We were able to assign age-class and cohort membership for 1,595 captures of 1,453 individuals (847 captures of 769 males, 748 captures of 684 females) for our analysis of growth in animals of known age. Of these, most were assigned to age-class 0 (52 males, 36 females) or age-class 1 (702 males, 652 females), with decreasing numbers assigned to subsequent age classes (25, 15, 19, 7, 17, 3, 6, and 1 males and 12, 6, 9, 9, 9, 6, 6, and 3 females were assigned to age-classes 2–9, respectively). We also obtained growth increments based on captures in successive years for 1,152 males and 730 females for our analysis of growth in animals of unknown age.

Members of age-class 0 appeared not to grow prior to first hibernation. SVL was uncorrelated with capture day of year in this age-class (*r* = 0.003, *n* = 88, *P* = 0.977; Fig 2). Furthermore, the SVL of members of age-class 0 did not differ from members of age-class 1 captured early in the active season (independent samples t-test comparing SVL of 88 members of age-class 0 with 65 members of age-class 1 captured prior to May 28 (DOY = 148); mean SVL = 195 vs. 198 mm, t = -1.01, df = 151, P = 0.314; Fig 2). Consequently, members of age-class 0 were omitted from growth analyses. Growth by most members of age-class 1 was clearly evident. However, some individuals failed to thrive as indicated by SVLs similar to those of newborns (i.e., ≤ 225 mm) in mid-summer or later (Fig 2).

### Individual Variation in Asymptotic Size

To characterize individual variation in asymptotic size, we applied the three-parameter von Bertalanffy growth equation to individual animals captured in five or more years. We computed growth days between captures as described previously, treating the day of first capture as growth day 0. As with animals of unknown age, we fixed *k* to the sex-specific values obtained from our analysis of animals of known age. We then estimated *SVL*_*A*_ and *SVL*_*0*_ using an iterative non-linear regression procedure in IBM SPSS Statistics 21. Here, *SVL*_*0*_ represents an estimate of SVL on the day of first capture. Ninety-five percent confidence intervals for *SVL*_*A*_ were generated using bootstrap estimates of the standard error.

We identified 69 males and 71 females that were each captured in five to nine different years. We were able to fit the three parameter growth model with *k* constrained to 0.0026 (males) and 0.0017 (females) to all but one individual. Ninety-five percent confidence intervals around estimates of *SVL*_*A*_ were sometimes broad (up to 134 mm in males and 346 mm in females). We therefore excluded individuals for which confidence intervals were broader than 10% of *SVL*_*A*_, leaving 63 males and 34 females for further consideration. Similar results were obtained in analyses in which the growth constant *k* was allowed to vary but with fewer individuals meeting our criterion for inclusion (S1 Table).

### Asymptotic Size and Lifetime Reproductive Success

**Fig 3:**
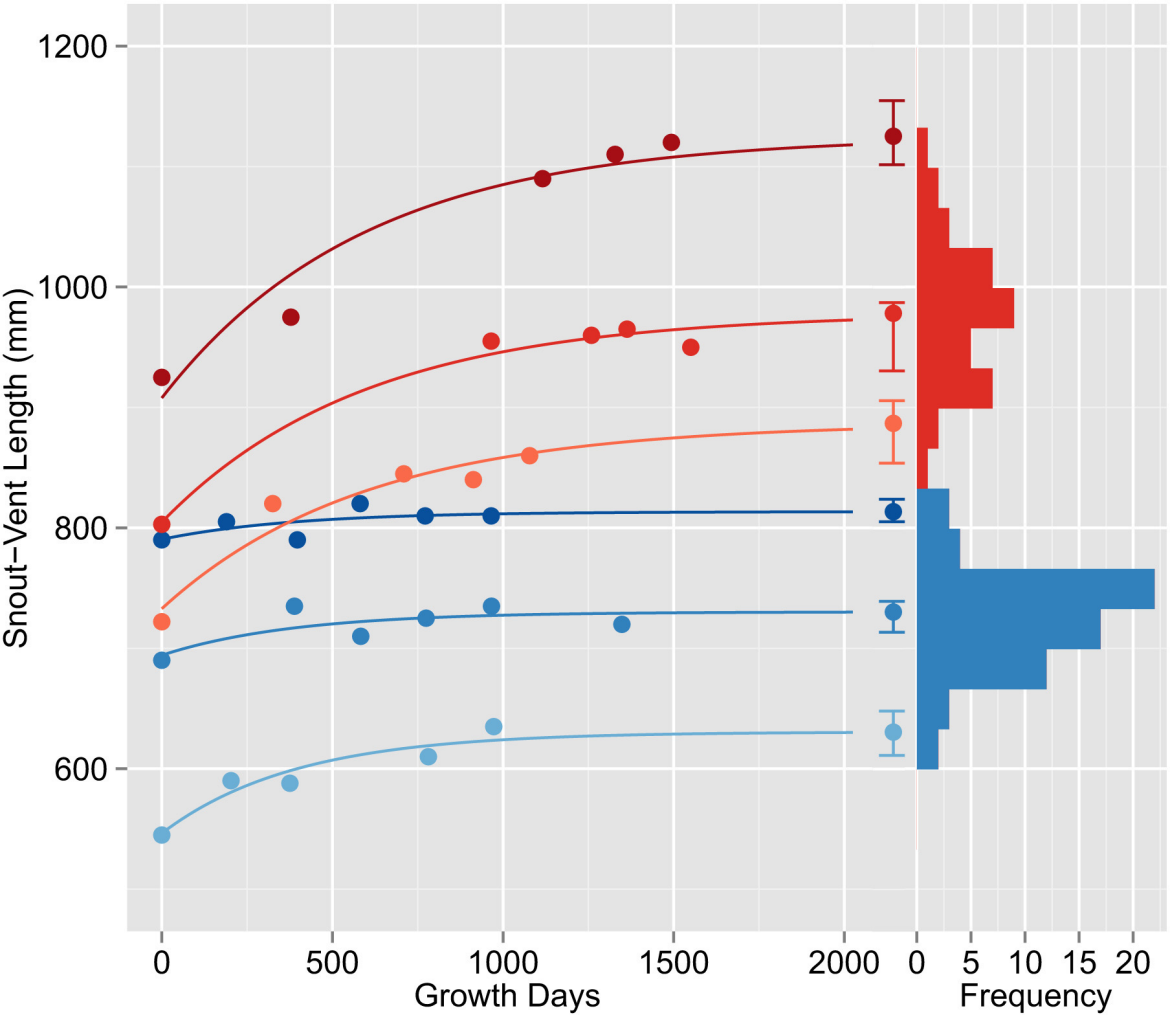
Representative individual growth trajectories (left) and distribution of individual asymptotic SVL of adult female (shades of red) and male (shades of blue) Lake Erie Watersnakes. Growth trajectories were plotted by setting the date of first capture to growth day 0 and fitting lines of the form *SVL*_*t*_ = *SVL*_*A*_ −((*SVL*_*A*_ − *SVL*_0_)*e*^−*kt*^) with *k* = 0.0017 for females and 0.0026 for males. Asymptotic SVL and 95% confidence intervals are indicated to the right of individual growth trajectories.

Perhaps the most apparent effect of variation in Lake Erie Watersnake body size is on offspring production by females [25]. To explore the potential impact of individual variation in asymptotic size, we conducted a simple thought experiment in which we compared estimated lifetime reproductive success among females with low (835 mm), intermediate (977 mm), and high (1125 mm) asymptotic SVL (See S1 File). We used the female SVL-fecundity relationship, ln(offspring #) = -12.2 + 2.29ln(female SVL) [25], and mean (0.73), minimum (0.63) and maximum (0.86) estimates of annual survival among 14 study sites [31]. We assumed that the probability of reproduction within a given year is 0.878 and uncorrelated with female size [32]. We further assumed that females follow the population mean growth trajectory until the onset of reproduction and individual-specific growth trajectories (Fig 3) thereafter. We computed lifetime reproductive success by summing annual offspring production, conditioned on adult survival and probability of reproduction. We considered two alternative scenarios regarding the onset of reproduction, one in which all females commence reproduction at 3 yr. 9 mo. of age and one in which reproduction is delayed by one or two years in females with intermediate or high asymptotic SVL, consistent with the expectation that growth might slow with the onset of reproduction.

## Results

### Population Mean Growth Trajectories

Our analysis of known age animals yielded population mean estimates for the growth constant, *k*, of 0.0026 for males and 0.0017 for females (Table 1). Although *k* was greater for males, the larger asymptotic size of females meant that growth rate was virtually indistinguishable between males and females among age-class 1 animals (Fig 2). Growth trajectories diverged among older age classes with females growing faster and achieving greater SVL than males (Fig 2).

**Table 1:** Population mean growth parameter estimates (95% confidence limits) for male and female Lake Erie Watersnakes based on analyses of animals of known and unknown age. In unconstrained analyses, all parameters were allowed to vary; in constrained analyses, some parameter values [brackets] were fixed. Least biased estimates of population mean *k* and *SVL*_*A*_ are shown in bold. Values of *SVL*_*0*_ obtained analytically are denoted with asterisks (*).

Sex	Data set and analysis	*n*	*k*	*SVL*_*A*_	*SVL*_*0*_	*r*^*2*^
Male	Known age, unconstrained	795	**0.0026**	714	53	0.949
			(0.0024–0.0027)	(697–730)	(43–62)	
	Known age, constrained	795	[0.0026]	[740]	44	0.946
					(42–46)	
	Unknown age, constrained	1152	[0.0026]	**740**	44*	0.854
				(736–744)		
	Unknown age, unconstrained	1152	0.0023	746	59*	0.855
				(741–752)		
Female	Known age, unconstrained	712	**0.0017**	1015	50	0.965
			(0.0016–0.0018)	(984–1046)	(41–58)	
	Known age, constrained	712	[0.0017]	[1018]	49	0.965
					(46–51)	
	Unknown age, constrained	730	[0.0017]	**1018**	51*	0.854
				(1007–1029)		
	Unknown age, unconstrained	730	0.0022	989	55*	0.860
				(977–1001)		

Using values of *k* from the analysis of animals of known age, our analysis of animals of unknown age yielded estimates of population mean asymptotic size, *SVL*_*A*_, of 740 mm for males and 1018 mm for females (Table 1, Fig 2). We repeated this analysis, without constraining *k*, and obtained somewhat different parameter estimates but equally high coefficients of determination (*r*^*2*^ from our constrained vs. unconstrained analysis = 0.854 vs. 0.855 for males and 0.854 vs. 0.860 for females; Table 1), suggesting that constraining *k* had a negligible effect on the fit of the resulting non-linear regression. Similarly, we repeated our analysis of animals of known age constraining both *k* and *SVL*_*A*_ and obtained coefficients of determination virtually identical to those of our unconstrained analysis (*r*^*2*^ from our constrained vs. unconstrained analysis = 0.946 vs. 0.949 for males and 0.965 vs. 0.965 for females; Table 1).

### Individual Variation in Asymptotic Size

**Fig 4:**
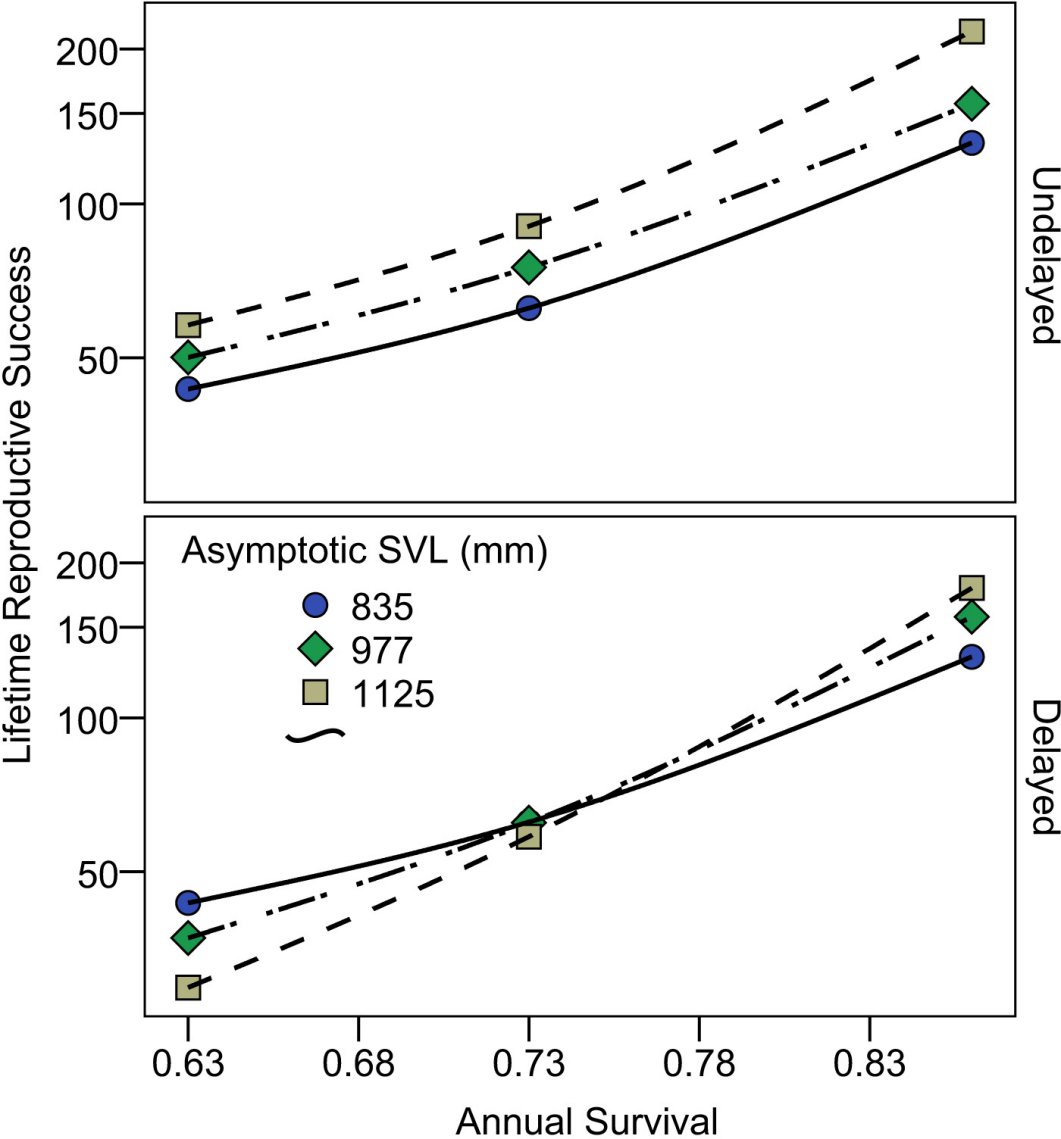
Predicted lifetime reproductive success of female Lake Erie Watersnakes as a function of annual survival and asymptotic SVL when reproduction is undelayed (upper panel; all females begin reproducing in year 4) and when reproduction is delayed by one or two years for females of average or high asymptotic size (lower panel). Symbol shape and line style distinguishes simulations with low (835 mm, circles and solid line), intermediate (977 mm, diamonds and dot-dash line) and high (1125 mm, squares and dashed line) asymptotic size.

Among individual males, *SVL*_*A*_ ranged from 631–820 mm (mean = 726 mm, standard deviation = 41.2 mm); among individual females, *SVL*_*A*_ ranged from 835–1125 mm (mean = 977 mm, standard deviation = 62.1 mm; Fig 3). In contrast to population mean growth trajectories, which give the impression of animals converging on a single asymptotic size (Fig 2), individual growth trajectories reveal wide variation in asymptotic size (by 189 mm among males and 290 mm among females; Fig 3). On average, 95% confidence intervals for asymptotic SVL spanned 39 mm for males (range = 12–77 mm) and 63 mm for females (range = 32–98 mm). Using overlap in 95% confidence intervals as an indicator of significance, asymptotic SVL of any given male differed from 50% of the other males analyzed (range = 21–92%) and asymptotic SVL of any given female differed from 53% of the other females analyzed (range = 19–97%). Asymptotic *SVL* of females consistently exceeded that of males (Fig 4). Furthermore, 95% confidence limits of males and females did not overlap except for one female whose lower limit overlapped the upper limit of 11 males.

To assess the closeness of individuals to their asymptotic size, we computed the expected annual growth increment for each individual given their current SVL (we used mean SVL of the two most recent captures as an estimate of current SVL to reduce measurement error). Among males, expected annual growth averaged just 3.1 mm and only two of 63 individuals exhibited expected annual growth greater than 10 mm (maximum = 18.0 mm). Among females, expected annual growth averaged just 5.4 mm and only five of 34 individuals exhibited expected annual growth greater than 10 mm (maximum = 17.5 mm).

### Asymptotic Size and Lifetime Reproductive Success

Female body size has a large impact on fecundity in Lake Erie Watersnakes with expected litter size nearly doubling when comparing a female measuring 835 mm (24.7 offspring) to one measuring 1125 mm (48.8 offspring) [25]. Consequently, when reproduction commences at the same age in all females, lifetime reproductive success was markedly greater for females with higher asymptotic size, regardless of annual survival (Fig 4, upper panel; See S1 File). However, when reproduction is delayed in females with intermediate and high asymptotic SVL, lifetime reproductive success was greatest for females with low asymptotic SVL when annual survival is low (0.63), was roughly equal among females when annual survival is intermediate (0.73), and was greater for larger females only when annual survival is high (0.86; Fig 4, lower panel; See S1 File).

## Discussion

Long-term study and large sample size allows for an unusually detailed analysis of Lake Erie Watersnake growth. The most striking result of our analyses is that asymptotic size varies dramatically among individuals. Consequently, population mean growth trajectories provide inaccurate representations of individual growth, especially for older, larger individuals. Although this problem has been noted previously, particularly for the difficulty it creates in estimating age from size [33]), the extent of individual variation has rarely been documented. Furthermore, individual variation in asymptotic size of Lake Erie Watersnakes appears to exceed that of other reptiles. Using the difference in extremes of asymptotic size relative to the midpoint as an index of variation, asymptotic size of Lake Erie watersnakes varies by 26% (males) and 30% (females) compared to 7% (males) and 8% (females) in Snapping Turtles [34]), 16% (males) and 19% (females) in Australian Freshwater Crocodiles (slow vs. rapidly growing population segments, [17]), 20% in Green Sea Turtles [19], and 25% in Meadow Vipers (estimated from Fig 1 in [16]). Among *Anolis* lizards, the coefficient of variation in SVL among animals that have ceased growth was less than 4% [35]. One practical implication of this variation is that shortcuts for estimating population mean asymptotic size (e.g., the largest 1, 10, or 10% of individuals sampled; [26, 36]), which may be appropriate in species with little variation in asymptotic size [35], are unlikely to be useful in species like Lake Erie Watersnakes.

One frequent use of population mean growth trajectories is to estimate age at sexual maturity. Based on a minimum SVL at sexual maturity of 460 mm in males (smallest male with sperm evident in cloacal smears) and 665 mm in females [32], we estimate that, on average, males reach sexual maturity after ca. 370 growth days or in September of their second year of life (chronological age ≈ 2 yr. 1 mo.) and females after ca. 600 growth days or in May of their fourth year of life (3 yr. 9 mo.). Because mating occurs in May and June and parturition occurs in late August and September [1]), we estimate that males average three years of age and females average four years of age when their first offspring are produced. However, if the variation we see in asymptotic size is associated with variation in the onset of sexual maturity, some Lake Erie Watersnakes may first produce offspring at younger or older ages.

Although larger females have greater fecundity, our estimation of lifetime reproductive success suggests that if greater asymptotic size requires that reproductive maturity be delayed, females achieving lower asymptotic size may be at an advantage if adult annual survival is low. While this relationship between growth, survival, and lifetime reproductive success is expected on theoretical grounds [37, 38], the fact that we can show advantages to alternative growth trajectories and asymptotic SVL using realistic parameter estimates suggests real-world significance. Differences in survival and adult body size between hunted and un-hunted populations of the Japanese Mamushi Snake [39]) and between Maryland and Ontario populations of Black Rat Snakes [40] parallel the results of the thought experiment described here.

We urge caution in the interpretation of lifetime reproductive success scenarios until underlying assumptions can be tested. In particular, we note that information on how females differing in asymptotic size might also differ in juvenile growth rate, age of maturity, and female size-offspring number relationship is needed. A contrasting pattern of life history variation, in which large asymptotic size is associated with lower adult survival and vice versa, is seen in ecotypes of the Western Terrestrial Gartersnake, *Thamnophis elegans* [12]. Lakeshore populations exhibit larger *SVL*_*A*_ (>650 mm) and lower annual survival (<0.57) whereas meadow populations exhibit lower *SVL*_*A*_ (ca. 550 mm) and higher annual survival (>0.71). These differences are associated with continuous (lakeshore) vs. variable (meadow) prey availability and correspondingly high vs. low juvenile growth rates, accelerated (2.5 yr.) vs. delayed (4.5 yr.) sexual maturity, and elevated vs. depressed size-specific fecundity [13, 41]. Unlike Western Terrestrial Gartersnakes, in which ecotypes occupy different, geographically separated habitats, Lake Erie Watersnake life history variants co-occur within populations. Consequently, resource availability should be similar among individuals, lending plausibility to our assumption of similar juvenile growth rates and size-specific fecundities.

In addition to its effect on female fecundity, there are a number of other potential impacts of variation in asymptotic size. Males might also gain in fecundity with increasing SVL, both because of an increased probability of mating [42] and positive size-assortative mating [43]. Thus, a relationship among asymptotic size, survival and fecundity, similar to what we proposed above for females, might exist in males. Unfortunately, we lack relevant data on mating success to assess relative benefits of varying asymptotic size in male Lake Erie Watersnakes. Body size is also expected to influence prey size, survival, and vulnerability to predators in both males and females [1, 32, 44].

Of equal interest to the effects of variation in asymptotic size discussed above are its proximate causes. Possibly, variation in size at birth results in differing growth trajectories among individuals that translate into differing asymptotic SVL [45]. Among 3,776 live-born offspring from 142 litters born to wild-caught females between 2003 and 2009, SVL averaged 189.7 mm and 95% of offspring had SVL between 165–210 mm (lower and upper 2.5% were trimmed to exclude outliers). Adjusting growth functions so that SVL = 165 or 210 (instead of 189.7) on growth day 92 yields values of *SVL*_*A*_ of 625 and 840 mm for males and 850 and 1163 mm for females. These match closely the lower and upper limits of *SVL*_*A*_ obtained from our analysis of individual variation in asymptotic size (631–820 mm in males and 835–1125 mm in females) and suggest that at least some individual variation in asymptotic size could be attributable to variation in size at birth. If so, tradeoffs between offspring size and number [46] could contribute to the variation in asymptotic size we see. However, without corroborating evidence from individual growth trajectories of young snakes, as in [45], such an interpretation is premature.

In addition, differences in environmental conditions may produce cohort-specific variation in growth and size. Age-class 1 Lake Erie Watersnakes exhibit growth trajectories (slope of regression of SVL on date of capture) that differ significantly among year-site combinations [47]. The magnitude of this variation (up to two fold differences in daily growth rate) has the potential to accelerate or delay attainment of sexual maturity by as much as two years [47] and could easily contribute to the variation in asymptotic size reported here. However, as with asymptotic size itself, the proximate mechanisms responsible variation in juvenile growth trajectories remain elusive. Consistent year or site effects seem to be lacking, but data are limited to eight year-site combinations, rendering statistical analyses weak [47]. Among Water Pythons (*Liasis fuscus*), year-to-year variability in resource availability (dusky rat density) results in a ‘silver spoon’ effect in which animals born in years with high rat density show accelerated growth rates throughout life [15]. While the abundance of Lake Erie Watersnake prey (primarily round gobies, *Neogobius melanostomus* [48, 49]) may be less variable than dusky rats, low water temperature may restrict spring-time foraging opportunities [50], resulting in weather-induced year-to-year variation in access to prey. Weather-induced variation in ovulation date generates cohort effects on neonatal growth in other New World natricine snakes [51] and year-to-year variation in offspring condition generates cohort effects on neonatal growth in Meadow Vipers (*Vipera ursinii*; [16]). Whether such cohort effects contribute to the variation asymptotic size of Lake Erie Watersnakes is unknown.

It is also possible that some of the variation we see in asymptotic size reflects differences among study sites. Our analysis of individual variation in asymptotic size includes individuals from 12 study sites separated by up to 19 km. Gene flow occurs frequently among sites but local adaptation (e.g., in color pattern) is evident [52–54]. Furthermore, differences in annual adult survival among sites [31]) might favor corresponding differences in asymptotic size. Unfortunately, data are too few from most study sites to test this possibility. Of 63 males and 34 females included in our analysis of individual variation in asymptotic size, 31 males and 20 females came from a single study site, South Bass Island State Park, with 8 or fewer males and 6 or fewer females from other sites. However, even among South Bass Island State Park animals, variation in asymptotic size is high. *SVL*_*A*_ ranges from 631–765 mm in males and from 835–1042 mm in females compared to 631–820 mm in males and 835–1125 mm in females across all sites, suggesting that site effects alone do not explain the variation we observed. Possibly, selection on asymptotic size imposed by differences in survival among sites, coupled with frequent gene flow, contributes to high within-site variation in asymptotic size.

Finally, the degree to which variation in asymptotic size is heritable is unknown. Common garden experiments demonstrate a genetic basis for ecotypic differences in life history between lakeshore and meadow dwelling Western Terrestrial Gartersnakes [41]). Furthermore, litter differences in morphology (including offspring size), life history, behavior, and physiology attributable to genetic and maternal effects are well documented in snakes (reviewed by [55]; also see [39, 56, 57]).

Another novel result of our analysis is that growth by neonates appears to be delayed until after emergence from first hibernation. This contrasts with the pattern seen in some other New World natricines (e.g., *Thamnophis sirtalis*, *Storeria dekayi*, *S*. *occipitomaculata*), in which neonates grow rapidly during late summer and fall, prior to hibernation ingress [47, 51]. In this regard, neonatal Lake Erie Watersnakes may resemble neonatal Meadow Vipers which are born in the fall but delay feeding and growth until the following summer [16].

The analyses presented here raise as many questions as they answer. As noted earlier, further information is needed regarding juvenile growth rates, age of maturity, and female size-offspring number relationships for females differing in asymptotic size. Information is also needed on how these variables differ among years and sites and how the onset of reproduction affects subsequent growth. Such information would allow for more meaningful assessments of the fitness consequences of variation in asymptotic size and may be useful in population projections (e.g., population viability analysis). Increased numbers of individuals with long-term capture histories, particularly from sites other than South Bass Island State Park, would allow formal tests for differences in asymptotic size among sites as might be expected from observed differences in annual survival. Complete individual growth trajectories from birth to adulthood would provide information on the ontogeny of variation in asymptotic size and allow analysis of individual variation in the growth constant *k* in addition to *SVL*_*A*_.

## Supporting Information

S1 Fig Sample scatterplot showing the relationship between SVL and capture day of year (DOY) in 2003. Only animals ≤ 500 mm SVL are shown. Separate scatterplots were created for each year to identify members of age-class 0 (blue), members of age-class 1 (green), and individuals of unknown age (gray).
(EPS)

S2 Fig Growth of male (squares) and female (circles) Lake Erie Watersnakes of unknown age. Shown is the size of individual watersnakes at first capture (Year 1 Snout-vent Length) vs. size when captured the following year (Year 2 Snout-vent Length). Solid (males) and dashed (females) lines represent linearized versions of composite Von Bertalanffy growth functions, based on parameter estimates from analyses of animals of both known and unknown age (males: *SVL*_2_ = 740 −((740 − *SVL*_1_)*e*^−0.0026*195^); females: *SVL*_2_ = 1018 −((1018 − *SVL*_1_)*e*^−0.0017*195^)).
(EPS)

S1 File Spreadsheet for the calculation of lifetime reproductive success of females with differing asymptotic SVL and annual survival when reproduction is undelayed (all females begin reproducing in year 4) and when reproduction is delayed by one or two years for females of average or high asymptotic size. (XLSX)

S1 Table Similarity in mean, standard deviation, and range of SVL_A_ (in mm) when the growth constant k is fixed (to 0.0026 in males and 0.0017 in females; Analysis 1), when k is constrained to fall within a specified range (0.0012–0.0050 in males; 0.0008–0.0034 in females; Analysis 2), and when k is unconstrained (Analysis 3). Correlations between estimates of SVL_A_ from different analyses were high (Analysis 1 vs. 2: r = 0.974 for males and 0.971 for females; Analysis 1 vs. 3: r = 0.879 for males and 0.847 for females). However, because three (rather than two) parameters are estimated in Analysis 2 and 3, confidence intervals around individual SVL_A_ estimates were broader, fewer individuals met our criterion for inclusion (breadth of the confidence interval ≤ 10% of SVL_A_). For this reason, we present only the results of Analysis 1.
(DOCX)
