# Supplementary material for: Size Matters: Individual Variation in Ectotherm Growth and Asymptotic Size
Source: PLoS One. 2016 Jan 5;11(1):e0146299. doi: 10.1371/journal.pone.0146299 (PMC4712130; doi:10.1371/journal.pone.0146299)
Supplement: S1 Table — Correlations between estimates of SVLA from different analyses were high (Analysis 1 vs. 2: r = 0.974 for males and 0.971 for females; Analysis 1 vs. 3: r = 0.879 for males and 0.847 for females). However, because three (rather than two) parameters are estimated in Analysis 2 and 3, confidence intervals around individual SVLA estimates were broader, fewer individuals met our criterion for inclusion (breadth of the confidence interval ≤ 10% of SVLA). For this reason, we present only the results of Analysis 1. (DOCX) [file pone.0146299.s004.docx]

|  | Males | | | | |  | Females | | | | |
| --- | --- | --- | --- | --- | --- | --- | --- | --- | --- | --- | --- |
|  | k | Mean SVL_A_ | Standard Deviation | Range | # meeting criterion |  | k | Mean SVL_A_ | Standard Deviation | Range | # meeting criterion |
| Analysis 1 | 0.0026 | 726 | 42 | 631-820 | 63 |  | 0.0017 | 973 | 63 | 833-1125 | 34 |
| Analysis 2 | 0.0012-0.0050 | 727 | 41 | 631-814 | 51 |  | 0.0008-0.0034 | 949 | 65 | 823-1135 | 25 |
| Analysis 3 | unconstrained | 739 | 49 | 645-821 | 34 |  | unconstrained | 959 | 78 | 821-1128 | 19 |
